# Increased Excitability of Lateral Habenula Neurons in Adolescent Rats following Cocaine Self-Administration

**DOI:** 10.1093/ijnp/pyu109

**Published:** 2015-02-26

**Authors:** Peter A. Neumann, Masago Ishikawa, Mami Otaka, Yanhua H. Huang, Oliver M. Schlüter, Yan Dong

**Affiliations:** Neuroscience Department (Drs Neumann, Ishikawa, Otaka, and Dong), and Department of Psychiatry, University of Pittsburgh, Pittsburgh, PA (Dr Huang); Molecular Neurobiology, European Neuroscience Institute, Göttingen, Germany (Dr Schlüter).

**Keywords:** addiction, cocaine, lateral habenula, plasticity, membrane excitability

## Abstract

**Background:**

The lateral habenula is a brain region that has been critically implicated in modulating negative emotional states and responses to aversive stimuli. Exposure to addictive drugs such as cocaine negatively impacts affective states, an effect persisting longer than acute drug effects. However, the mechanisms of this effect are poorly understood. We hypothesized that drugs of abuse, such as cocaine, may contribute to drug-induced negative affective states by altering the firing properties of lateral habenula neurons, thus changing the signaling patterns from the lateral habenula to downstream circuits.

**Methods:**

Using whole-cell current-clamp recording of acutely prepared brain slices of rats after various periods of withdrawal from cocaine self-administration, we characterized an important heterogeneous subregion of the lateral habenula based on membrane properties.

**Results:**

We found two major relevant neuronal subtypes: burst firing neurons and regular spiking neurons. We also found that lateral habenula regular spiking neurons had higher membrane excitability for at least 7 days following cocaine self-administration, likely due to a greater membrane resistance. Both the increase in lateral habenula excitability and membrane resistance returned to baseline when tested after a more prolonged period of 45 days of withdrawal.

**Conclusion:**

This is the first study to look at intrinsic lateral habenula neuron properties following cocaine exposure beyond acute drug effects. These results may help to explain how cocaine and other drugs negatively impact affect states.

## Introduction

Drug addiction involves complex neural circuits and an enormous number of cellular and molecular adaptations. Acute exposure to drugs of abuse often elicits an emotional “high” while also inducing negative/aversive effects that outlast the initial positive feelings (Solomon and Corbit, 1974; Koob and Le Moal, 1997). This reduction in affect—one’s cognitive emotional state—by increasing negative emotional states and/or reducing positive emotional states may contribute to continued or chronic drug use as the user seeks to alleviate these negative feelings (Solomon and Corbit, 1974; Solomon, 1980; Koob and Le Moal, 1997). Chronic drug use is often a serious condition, as maladaptive emotional and motivational states can be developed, leading to compulsive drug use or addiction (Solomon, 1980; Koob and Le Moal, 2008). The opponent process theory posits that these prolonged aversive effects work in opposition to the positive rewarding effects of addictive drugs and that these aversive effects are also one of the major difficulties in abstaining from drug use, as users seek drugs in order to mitigate chronic negative affect (Solomon and Corbit, 1974; Solomon, 1980; Koob and Le Moal, 1997, 2001, 2008).

The lateral habenula (LHb) has recently garnered interest for its role in mediating negative rewards and aversive effects (Matsumoto and Hikosaka, 2007) and negative affect (Yang et al., 2008; Li et al., 2011). LHb neuronal activity is negatively correlated with neuronal activity in positive reward-related regions such as the ventral tegmental area (VTA) (Christoph et al., 1986; Ji and Shepard, 2007; Stamatakis et al., 2013). Additionally, direct application of cocaine to brain slices excites LHb neurons (Good et al., 2013; Zuo et al., 2013), while cocaine exposure has been shown to increase aversive conditioning via the LHb (Jhou et al., 2013).

The LHb is a heterogeneous region and includes several smaller subnuclei with physiology, connectivity, and functionality that are currently poorly defined (Andres et al., 1999; Weiss and Veh, 2011; Aizawa et al., 2012). Inputs to the LHb arrive from a variety of brain regions and include TH-positive projections from the VTA, indicating that dopaminergic neurons from the VTA send signals to the LHb (Lecourtier and Kelly, 2007; Hikosaka, 2010; Aizawa et al., 2012; Good et al., 2013). Outputs from the LHb are chiefly glutamatergic and primarily target the VTA and the rostromedial tegmental nucleus (RMTg), which then sends GABAergic signals to the VTA (Ji and Shepard, 2007; Lecourtier et al., 2008; Balcita-Pedicino et al., 2011; Matsui and Williams, 2011; Stamatakis et al., 2013). It appears, then, that the LHb is well-situated to mediate negative affect and aversive behaviors by controlling inhibitory signaling to VTA dopamine neurons via this LHb-to-RMTg-to-VTA pathway (Sesack and Grace, 2010; Balcita-Pedicino et al., 2011).

These circuitry-based reports position the LHb as a critical region for regulating drug-induced negative affect as well. Indeed, there is evidence that cocaine exposure induces synaptic plasticity specifically in the LHb-to-RMTg pathway (Maroteaux and Mameli, 2012), likely leading to increased inhibitory signaling from the RMTg to the VTA. Other studies have also examined the LHb-RMTg-VTA pathway and found supporting evidence for its involvement in cocaine-induced aversive behaviors (Jhou et al., 2013). However, it remains largely unknown whether exposure to drugs of abuse, such as cocaine, reshape or induce plastic changes within LHb neurons directly. Here, we demonstrate that short-term withdrawal (1–2 days) from cocaine self-administration results in significantly increased intrinsic membrane excitability and membrane resistance of LHb neurons. Given the highly regulated conditions under which LHb neurons operate, this adaptation may significantly increase the response of LHb neurons to incoming signals, thus contributing to increases in LHb signaling to downstream targets such as the RMTg and potentially contributing to the prolonged increases in negative affect following cocaine exposure. The cocaine-induced increase in LHb membrane excitability was maintained for at least 7 days after cocaine self-administration, but when measured after long-term withdrawal of 45 days, this membrane adaptation had returned to baseline levels. These results indicate that there may be a window of time following exposure to cocaine whereby LHb neurons exhibit cocaine-induced increases in excitability and downstream signaling.

## Methods

### Animals

Upon arrival, male Sprague-Daley Rats (Charles River) weighing 90 to 110g were housed in pairs with 12-h/12-h light/dark cycles and free access to food and water. Animals were allowed to habituate to their cages for at least 6 days before undergoing any procedures. Following catheter surgery, animals were single-housed. All experimental procedures were approved by the Institutional Animal Care and Use Committee of the University and were performed in accordance with the guidelines of the National Institutes of Health.

### Catheter Surgery

After habituating to their cages, p33-40 rats (weighing between 125 and 150g) underwent self-administration catheter surgery. Briefly, rats were anesthetized with a ketamine/xylazine mixture (50–100/5–10mg/kg, i.p.). A silicone catheter (0.51 inner/0.940-mm outer diameter, HelixMark) was then inserted into the jugular vein and run under the skin to a small incision made between the scapulae where it exited the body and connected to a harness with quick connect luer (SAI infusion technologies) worn by the rat. Throughout the recovery and training period, catheters were flushed daily through the harness with sterile saline solution containing gentamicin (5mg/mL) and heparin (10 USP/mL).

### Self-Administration Training

Following surgery, the rats were placed back in cages to be single-housed and allowed to recover for 7 to 12 days before beginning self-administration training. Self-administration operant chambers (Med Associates) contained 2 separate nose-poke holes 6cm above the grid floor. The harness luer was attached to a swivel with a tether and connected to a syringe loaded into an infusion pump. Nose pokes to the active hole initiated an infusion “reward” of cocaine/saline (0.75mg/kg cocaine or an equivalent volume of saline during 6 seconds; the volume of each infusion was 90–150 µL and was adjusted based on the body weight of the animal at each training session to meet this criteria), turned off the house light for 20 seconds, and turned on a separate 6-second cue light. A successful infusion was followed by a 14-second lockout period. During this lockout period, active nose pokes continued to be recorded but failed to initiate any cues or further infusions. At the end of this lockout period, the house light would turn back on, signaling that the active nose poke hole could now initiate another infusion. Nose pokes in the inactive hole were recorded but elicited no effects. All tests were done using an FR1 schedule.

Rats were randomly divided into cocaine and saline groups for across the 3 chosen withdrawal time points and were placed in the operant chambers for one overnight training session (approximately 12 hours) 8 to 10 days after surgery. Rats in a cocaine group that failed to receive >30 cocaine infusions during the overnight session were excluded from further testing (approximately 10% of the group). Approximately 24 hours later, rats began a series of daily 2-hour training sessions over 5 consecutive days. Rats in a cocaine group that did not show the ability to distinguish between active and inactive nose pokes or did not receive at least 15 cocaine infusions per 2-hour session were excluded from further study (approximately10% of the group). At the conclusion of the training, rats from all groups were placed back into their home cages for a 24- to 48-hour, 5- to 7-day, or a 43- to 47-day (referred to as 45 days) withdrawal period before being taken for brain slice preparation.

### Brain Slice Preparation

Following the withdrawal period, rats were quickly anesthetized with approximately 3 to 4mL of 99.9% isofluorane in a closed 20x16x16cm chamber and were decapitated so that the brain could be extracted. Coronal brain slices (260 µm thick) containing the LHb were prepared using a VT1200S microtome (Leica). Slices were cut in the presence of 4°C cutting solution containing (in mM): 135 *N*-methyl-d glucamine, 1 KCl, 1.2 KH_2_PO_4_, 0.5 CaCl_2_, 1.5 MgCl_2_, 20 choline-HCO_3_, and 11 glucose, saturated with 95% O_2_/5% CO_2_, with the pH adjusted to 7.4 using HCl, at 300 to 310 mOsm. After being cut, slices were placed in an incubation chamber in artificial cerebrospinal fluid containing the following (in mM): 119 NaCl, 2.5 KCl, 2.5 CaCl_2_, 1.3 MgCl_2_, 1 NaH_2_PO_4_, 26.2 NaHCO_3_, and 11 glucose, at 290 to 294 mOsm, saturated with 95% O2/5% CO_2_ to maintain a pH of 7.4 at 37°C for 30 minutes. Slices were then allowed to recover for at least 30 minutes at room temperature before being used for experimentation.

### Electrophysiological Recordings

Whole-cell current-clamp recordings were made in the LHb, specifically in the parvocellular and central parts of the medial division of LHb (Figure 2a). During recordings, slices were superfused with artificial cerebrospinal fluid that was heated to 31 to 33°C by passing the solution through a feedback controlled in-line heater (Warner Instruments) before entering the recording chamber. Recordings were made under visual guidance (40x, differential interference contrast optics) with micropipettes (2.5–5 MΩ) filled with a potassium-based internal solution containing (in mM): 130 KMeSO_3_, 10 KCl, 0.4 ethylene glycol tetraacetic acid, 10 4-(2-hydroxyethyl)-1-piperazineethanesulfonic acid, 2.5 Mg-ATP, 0.25 Na-GTP, and 2 MgCl_2_-6H_2_O, pH 7.3, 294 mOsm.

The holding current for each cell was adjusted such that the cell maintained a membrane potential of −65 mV, which is close to the average resting membrane potential of these neurons (Wilcox et al., 1988). Though rare, cells requiring currents greater than ±20 pA to reach this holding potential were excluded for data collection. Series resistance was 9 to 20 MΩ, uncompensated, and monitored continuously during recording. Recordings with a change in series resistance >20% for the duration of data collection were not accepted for data analysis. To record evoked action potential firing, current injection steps were generated using Clampex software (Molecular Devices). The range of −50 to +90 pA, 10 pA increments/steps, and 0.1 Hz/step was chosen based on calibration trials to find appropriate current steps that would elicit the best range of action potential spikes across the full sample of recorded LHb neurons. Voltage traces were recorded with a MultiClamp 700B amplifier, filtered at 3kHz, amplified 5 times, and then digitized at 20kHz. Three consecutive series of 10-pA steps (from −50 to +90 pA) were recorded from each cell. Cells were allowed approximately 5 minutes to stabilize after achieving the whole-cell patch configuration before data collection began.

### Drugs and Reagents

Cocaine-HCl was supplied by the Drug Supply Program of the National Institute of Drug Abuse. All other chemicals were purchased from Sigma-Aldrich.

### Statistics and Data Analysis

All cell measurements were averaged across 3 consecutive trials. The number of action potentials evoked by each current injection step was used as a measure of cell membrane excitability. If the number of peaks at any current step varied more than 20% across any of the 3 trials, the cell was excluded from analysis for being unstable. Membrane resistance measurements for each cell were calculated using Ohm’s law by taking the difference in the cell’s voltage between the final 100ms of each negative current step compared to the cell’s baseline and dividing by the amount of current injected. The threshold for action potentials was measured as the point at which the voltage level of the cell slopes upwards at >25 mV/ms during the final rise to form an action potential peak. Using this threshold as a baseline, the fast-decaying afterhyperpolarization (fAHP) was measured as the lowest point 2 to 5 milliseconds after the peak of the action potential, and the medium-duration afterhyperpolarization (mAHP) was measured as the lowest point 20 to 40 milliseconds after the peak of the action potential. Neurons that did not demonstrate either a clear fAHP or mAHP phase were excluded from analysis of that phase. All measurements were taken from the same set of recorded cells with none being excluded, unless specifically stated. Student’s *t* test was used to compare single data points, while 2-way repeated measures analysis of variance was used to compare treatment groups across multiple data points (spike numbers across all current steps and treatment groups, or behavioral data across multiple days and treatment groups). Results are shown as mean ±SEM.

## Results

### Self-Administration of Cocaine or Saline

**Figure 1.**
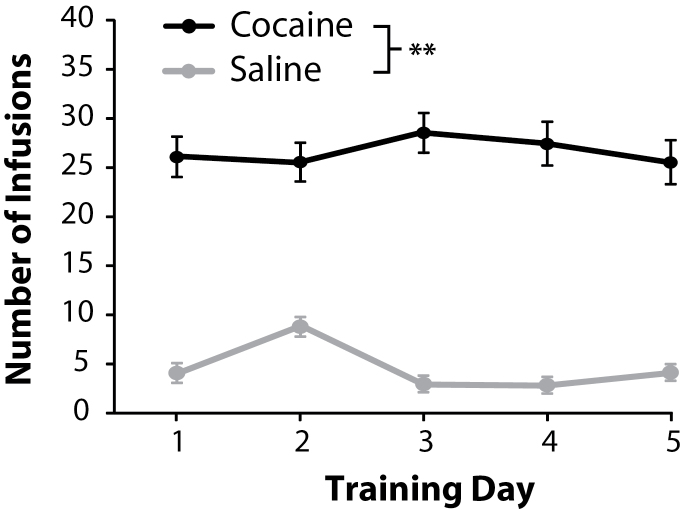
The self-administration protocol leads to cocaine-seeking behavior in rats. Rats receiving cocaine nose-poked for more infusions than rats receiving saline. The graph shows the average number of infusions for rats in cocaine groups and saline groups across the 5 daily 2-hour self-administration training sessions. Data from short-term (ST), moderate-term (MT), and long-term (LT)withdrawal groups were combined (n of animals per treatment condition: saline ST, 6; MT, 4; LT, 3; total = 13; cocaine ST, 6; MT, 6; LT, 3; total = 15).

To test the effects of cocaine exposure on LHb neurons, a self-administration training model was used. Rats received 5-day self-administration training after an initial overnight training session. Rats were trained to nose-poke for 0.75mg/kg infusions of cocaine/saline during 2-hour sessions. Animals in short-term (ST), moderate-term (MT), and long-term (LT) withdrawal groups received identical training and access to cocaine/saline. ANOVA was used to compare the number of rewards received across all treatment groups and training days, whereby reward infusions was the dependent variable and the training day and treatment group were fixed factors. As expected, comparisons between the ST, MT, and LT withdrawal groups for rats revealed no differences between reward infusions after a Bonferroni posttest (ST vs LT: saline, *P* =1.00, n = 6, 3; cocaine, *P* =1.00, n = 6, 3; ST vs MT: saline, *P* =1.00, n = 6, 4; cocaine, *P*=1.00, n = 6, 6; MT vs LT: saline, *P* =1.00, n = 4, 3; cocaine, 1.00, n = 6, 3). Thus, the behavioral results were combined across withdrawal groups. Analysis comparing saline and cocaine treatment groups revealed that rats in combined cocaine treatment groups nose poked for more infusions than rats in the combined saline group (Figure 1; *P* < .0001, F[1, 139] = 562.64, n coc/sal = 15/13). The individual day of training had no effect (*P*=0.69, F[4, 139] = 0.56, n coc/sal = 15/13), and no interaction effects were present across withdrawal groups or training days. These results confirm that this 5-day self-administration procedure is sufficient to both initiate and measure cocaine-seeking behavior in adolescent rats and that these rats demonstrate equivalent cocaine-seeking across withdrawal groups.

### Characterization of Two LHb Neuron Subtypes

**Figure 2.**
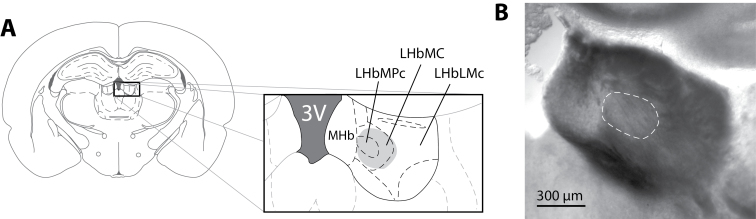
The lateral habenula (LHb) and subnuclei regions. (*a*) Diagram showing the parvocellular and central regions of the medial division of the LHb where cells were recorded (shaded region). 3V, third ventricle; LHbMPc, parvocellular part of the medial division of the LHb; LHbMC, central part of the medial division of the LHb; LHbLMC, magnocellular part of the lateral division of the LHb. (*b*) Differential interference contrast image of the habenula of a rat brain slice. The LHb region of interest is apparent from natural markings in the surrounding areas and is outlined by a dashed white line in this picture.

At 24 to 48 hours following the fifth and final self-administration training session, rats were sacrificed to obtain coronal brain slices containing the LHb for whole-cell current-clamp recordings. Cells located in the parvocellular or central parts of the medial division of the LHb were preferentially targeted for recording (Figure 2a), as cells in these regions have been shown to receive the highest density of incoming TH-positive fibers originating from the VTA (Aizawa et al., 2012; Good et al., 2013). The target region was easily discernable under standard differential interference contrast optics (Figure 2b).

**Figure 3.**
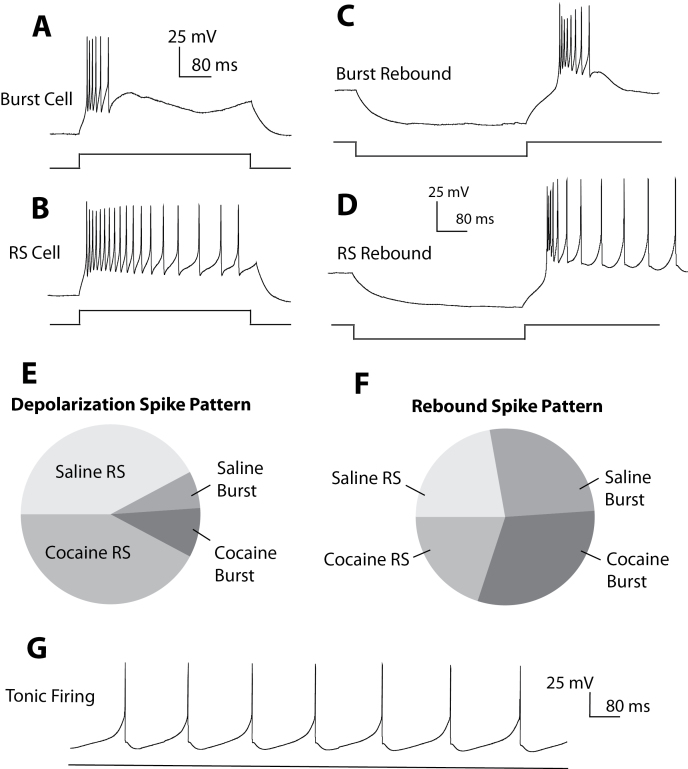
Cell types and spiking patterns in the LHb. (*a*) Example traces showing a typical burst firing (BF) pattern and (*b*) regular spiking (RS) pattern following depolarizing injections of current. (*c*-*d*) Example traces showing typical (*c*) BF and (*d*) RS rebound spiking patterns. (*e*) Chart showing the relative ratios of BF and RS spiking LHb cells during depolarization following saline (BF = 3, RS = 19, total = 22; 6 rats) or cocaine (BF = 4, RS = 19, total = 23; 6 rats) self-administration training. (*f*) Chart showing the relative ratios of BF and RS rebound spiking patterns in LHb cells following saline (BF = 12, RS = 10, total = 22; 6 rats) or cocaine (BF = 14, RS = 9, total = 23; 6 rats) self-administration training. (*g*) Example trace showing a typical approximately 5-Hz tonic firing pattern from an LHb cell at rest.

A survey of LHb cells revealed 2 relevant major neuronal types: burst firing (BF) cells and regular spiking (RS) cells (Figure 3a-b). Continued observation revealed that 3 of 22 recorded cells from saline-treated animals showed clear BF characteristics, while 4 of 23 recorded cells from cocaine-treated animals showed clear BF characteristics (Figure 3e), indicating that cocaine exposure does not shift the population composition of BF/RS cell types in the LHb.

All recorded LHb neurons also exhibited action potentials evoked by releasing a hyperpolarizing stimulation, termed rebound spiking (Wilcox et al., 1988; Chang and Kim, 2004; Weiss and Veh, 2011) (Figure 3c-d). Similar to depolarization spiking in LHb neurons, rebound spiking occurs in trains of RS or BF patterns. These rebound spike trains continue for various extended periods of time depending upon the magnitude of the preceding hyperpolarization. Interestingly, the rebound spiking pattern did not necessarily match the depolarization spiking pattern within the same cell, even at the same holding potential. Whereas only 3 of 22 cells recorded from saline-treated animals exhibited BF spike patterns during depolarization, 12 of the 22 recorded cells showed BF spike patterns following hyperpolarization (Figure 3e-f). Similarly, while only 4 of 23 recorded cells from cocaine-treated animals had BF spike patterns during depolarization, 14 of these 23 cells had BF rebound spike patterns (Figure 3e-f). All cells that showed BF spiking during depolarization also showed BF rebound spiking. The similarity of these sample numbers between the saline and cocaine groups, again, indicates that cocaine exposure does not shift the population composition or resting potential of cell types within the LHb.

Upon achieving a whole-cell patch-clamp configuration, most LHb neurons demonstrated a variety of tonic firing patterns, ranging between 0.1 and 10 Hz (Figure 3g). This tonic firing was variable and unstable over time, though, and even disappeared in many cases approximately 10 to 20 minutes after cells came to rest. However, continued persistence or even intensification in other cases complicated certain measurements (such as rheobase current). Recorded neurons were injected with current (up to ±20 pA) in order to maintain a resting potential near −65 mV. A majority (approximately 85%) of LHb neurons demonstrated RS spiking patterns, while a minority (approximately 15%) demonstrated BF spiking patterns. It should also be noted that a relatively small amount of current is needed to bring these LHb neurons to their action potential threshold. The current steps used in the present study ranged from −50 to +90 pA, where spike numbers began to plateau in some cells. A majority of recorded LHb neurons fire multiple spikes with just a 400-millisecond injection of +10 pA, confirming that neurons in the LHb are highly regulated and sensitive to incoming signals (Wilcox et al., 1988; Chang and Kim, 2004; Weiss and Veh, 2011).

### Membrane Excitability of LHb Neurons Is Increased after Short-Term Withdrawal from Cocaine Self-Administration

**Figure 4.**
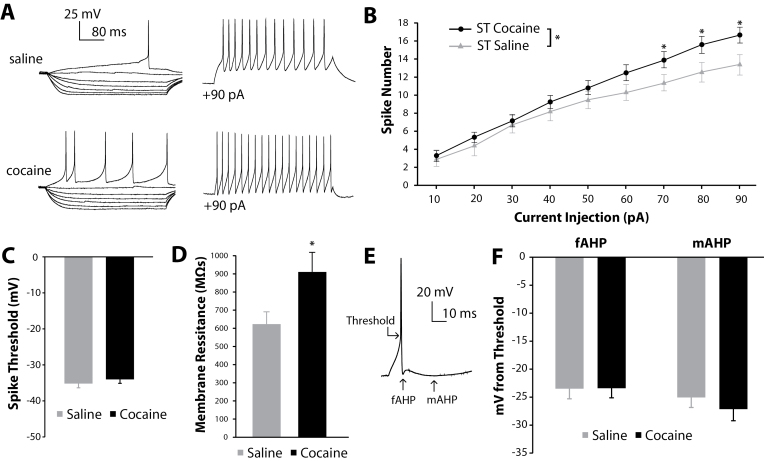
Lateral habenula (LHb) neuron characteristics 1 to 2 days after cocaine/saline self-administration. (*a*) Example traces showing typical current steps from −50 to +10 pA (left) and +90 pA (right) in LHb neurons after saline (top) or cocaine (bottom) self-administration. (*b*) Plot showing the mean number of spikes fired at each current step from LHb neurons 24 to 48 hours after cocaine or saline self-administration training (saline/cocaine, n = 19/19; rats = 6/6). (*c*) Graph showing the mean threshold of action potentials (saline/cocaine, n = 19/19; rats = 6/6). (*d*) Graph showing the mean input resistance of LHb cells (saline/cocaine, n = 19/19; rats = 6/6). (*e*) Example of fast-decaying afterhyperpolarization (fAHP) and medium-duration afterhyperpolarization (mAHP) measurement locations on a typical isolated spike trace. (*f*) Graph of mean fAHP (saline/cocaine, n = 10/8; rats = 6/6) and mAHP (saline/cocaine, n = 13/15; rats = 6/4) measurements relative to spike threshold. *, *P* < .05 based on ANOVA comparison in (*b*) and Ttest in (*d*).

To examine the impact of cocaine self-administration on the membrane excitability of LHb cells, we elicited action potentials from these neurons using a series of current injection steps (-50 to +90 pA, 10-pA increments) 24-48 hours after the final self-administration training session. Cells that demonstrated BF spiking during depolarization (approximately 15% of cells) were excluded from excitability analysis due to their irregular firing pattern. Spike numbers at each current step were counted as a measure of the membrane excitability (Figure 4a). Two-way repeated measures ANOVA with spike number as the dependent variable repeated at each current step for both saline and cocaine treatment groups revealed that animals from the treatment group had a significant effect on the membrane excitability in LHb cells 24 to 48 hours after the final cocaine exposure when compared to saline-exposed controls (Figure 4b, *P* < .05, F[1, 36] = 5.62, n = 38). Bonferroni’s multiple comparisons test was performed to detect significant differences between treatment groups at each current step.

We then looked to examine additional properties of these LHb neurons in an attempt to determine contributing factors to the observed cocaine-induced increase in the membrane excitability. We first measured the threshold of action potentials in single-standing spikes. There were no significant differences in the threshold for action potentials between saline- and cocaine-exposed rats (Figure 4c, *P*=0.48, n sal/coc = 19/19). We next calculated the membrane resistance of the LHb cells by measuring the cell’s change in potential in response to negative current injections (−10 to −50 pA). We found that the membrane resistance was increased in cocaine-treated rats compared with saline-treated rats (Figure 4d; *P* < .05, n sal/coc = 19/19). We also measured fast and medium components of AHPs (Figure 4e) and observed no differences in these 2 parameters between cocaine- and saline-treated groups (Figure 4f; fAHP, *P*=.91, n sal/coc = 13/16; mAHP, *P*=.49, n sal/coc = 9/6). Thus, an increase in membrane excitability is correlated with an increase in membrane resistance.

### Membrane Excitability of LHb Neurons Following Long-Term Withdrawal from Cocaine

**Figure 5.**
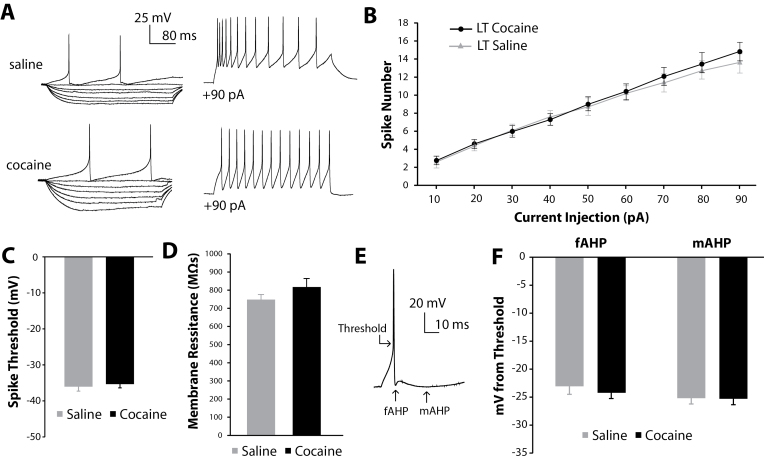
Lateral habenula (LHb) neuron characteristics 45 days after cocaine/saline self-administration. (*a*) Example traces showing typical current steps from −50 to +10 pA (left) and +90 pA (right) in LHb neurons after 45 days of withdrawal from saline (top) or cocaine (bottom) self-administration. (*b*) Plot showing the mean number of spikes fired at each current step from LHb neurons 45 days after cocaine or saline self-administration training (saline/cocaine, n = 10/15; rats = 3/3). (*c*) Graph showing the mean threshold of action potentials (saline/cocaine, n = 10/15; rats = 3/3). (*d*) Graph showing the mean membrane resistance of LHb cells (saline/cocaine, n = 10/15; rats = 3/3). (*e*) Example of fAHP and mAHP measurement locations on a typical isolated spike trace. (*f*) Graph of mean fAHP (saline/cocaine, n = 6/10; rats = 3/3) and mAHP (saline/cocaine, n = 7/12; rats = 3/3) measurements relative to spike threshold.

After observing that cocaine self-administration leads to an increase in the membrane excitability of LHb neurons 24 to 48 hours later, we then looked at a more protracted withdrawal time point at 45 days to determine if these changes were persistent. The same cell characteristics measured at 24 to 48 hours of withdrawal were then measured after 45 days of withdrawal. Again, spike numbers at each current step were counted as a measure of the membrane excitability (Figure 5a). Two-way repeated-measures ANOVA using spike number as the dependent variable repeated at each current step for cocaine LT and saline LT treatment groups revealed no significant effect of the treatment on the number of spikes across all current steps (F[1, 23] = 0.17, n = 25, *P*=.68). Thus, it appears that the initial cocaine-induced increase in membrane excitability returns to baseline levels at some time point after 48 hours of withdrawal (Figure 5b). Additionally, the membrane resistance no longer differed significantly from saline-exposed controls 45 days after the last cocaine exposure (Figure 5d). All other cellular measures including action potential threshold, fAHP, and mAHP also showed no statistical differences between treatment groups after LT withdrawal (Figure 5c, e, f). Taken together, these results indicate that the cocaine-induced increase in LHb cell excitability returns to baseline at some point between 2 and 45 days of withdrawal from cocaine and is correlated with changes in membrane resistance.

### Membrane Excitability of LHb Neurons Following Moderate-Term Withdrawal from Cocaine

**Figure 6.**
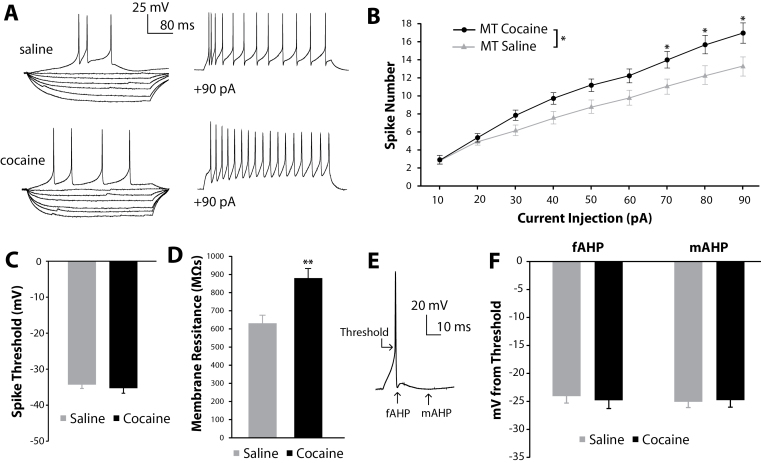
Lateral habenula (LHb) neuron characteristics 5 to 7 days after cocaine/saline self-administration. (*a*) Example traces showing typical current steps from −50 to +10 pA (left) and +90 pA (right) in LHb neurons after 5 to 7 days of withdrawal from saline (top) or cocaine (bottom) self-administration. (*b*) Plot showing the mean number of spikes fired at each current step from LHb neurons 5 to 7 days after cocaine or saline self-administration training (saline/cocaine, n = 18/9; rats = 4/6). (*c*) Graph showing the mean threshold of action potentials (saline/cocaine, n = 18/19; rats = 4/6). (*d*) Graph showing the mean membrane resistance of LHb cells (saline/cocaine, n = 18/19; rats = 4/6). (*e*) Example of threshold, fast-decaying afterhyperpolarization (fAHP), and medium-duration afterhyperpolarization (mAHP) measurement locations on a typical isolated spike trace. (*f*) Graph of mean fAHP (saline/cocaine, n = 15/13; rats = 4/6) and mAHP (saline/cocaine, n = 14/14; rats = 4/6) measurements relative to spike threshold. *, *P* < .05, based on ANOVA comparison; **, *P* < .01 based on *t* test.

After observing that the cocaine-induced increases in membrane excitability and membrane resistance returned to saline-control levels by day 45 of withdrawal, we decided to examine a 5- to 7-day moderate term (MT) withdrawal point to better understand the time course of these observed changes. Again, the same cell characteristics were measured at this MT time point as were measured at the ST and LT withdrawal time points. A 2-way repeated-measures ANOVA using spike number as the dependent variable repeated at each current step for saline MT and cocaine MT treatment groups showed that the cocaine MT treatment had a significant increase in cell excitability (F[1, 35] = 5.39, n = 37, *P* < .05), similar to the cocaine ST treatment group (Figure 6a, b). Bonferroni’s multiple comparisons test was used to check for differences at each current step. This result reveals that LHb neurons maintain increased levels of excitability until at least 7 days after cocaine self-administration training (a Bonferroni posttest comparing withdrawal days 5–7 showed no differences between withdrawal days; *P*=1.00 for all comparisons). Measurements of other cellular characteristics including the action potential threshold, fAHP, and mAHP again revealed no significant effects of cocaine at the MT withdrawal point (Figure 6c, e, f). However, the membrane resistance of LHb cells after MT withdrawal from cocaine self-administration was again significantly different from saline controls (Figure 6d; *p* < .01, n sal/coc = 18/19), further supporting a correlation between membrane excitability and membrane resistance.

## Discussion

The present study demonstrates that LHb RS neurons have increased membrane excitability 24 to 48 hours following self-administration of cocaine, which lasts until at least 7 days after the last cocaine exposure. There was no difference in the distribution of cell types, action potential threshold, or fast/medium duration hyperpolarization potentials within the LHb after cocaine self-administration. As an increase in membrane resistance was observed in correlation with the increase in excitability of LHb cells after ST and MT withdrawal, it seems likely that the observed increase in cell excitability is at least partially mediated by an increase in membrane resistance. This cocaine-induced increase in intrinsic excitability amplifies LHb neuron signal transmission. Upon receiving equivalent input, LHb neurons in cocaine-trained animals transmit a greater number of signals to downstream targets, enhancing the contribution of the LHb to the involved circuits. As the LHb appears to mediate negative affect and aversive behaviors, this amplification of LHb signaling may represent a drug-induced alteration contributing to the increased opponent processes and prolonged negative affect known to occur after cocaine and addictive drug use.

Early studies examining the functional and behavioral role of the LHb largely targeted the entire structure, treating it as a homogenous region. However, molecular and ultra-structural characterizations have revealed that the LHb is highly heterogeneous, with many potentially distinct subnuclei involved in specific circuits and functions (Andres et al., 1999; Weiss and Veh, 2011; Aizawa et al., 2012). The present study recorded from neurons located in the parvocellular and central subregions of the medial LHb, which receive the majority of TH-positive projections from the VTA (Aizawa et al., 2012; Good et al., 2013). LHb neurons that receive TH-positive fibers from the VTA primarily send glutamatergic projections to the RMTg (Balcita-Pedicino et al., 2011; Stamatakis et al., 2013). The receiving neurons in the RMTg then send inhibitory projections to VTA dopamine neurons (Christoph et al., 1986; Jhou et al., 2009; Omelchenko et al., 2009; Stamatakis et al., 2013). Thus, if signals coming out of the LHb are tonically amplified after cocaine exposure, VTA dopamine neurons receive a greater amount of tonic inhibition from the RMTg, causing less dopamine to be released in reward-related regions and reducing positive affect levels of an individual. This scenario supports the hypothesis that increased LHb neuron membrane excitability may contribute to the chronic increase in negative affect states following cocaine and addictive drug exposure.

Increased excitability is observed at least 7 days after the final cocaine exposure, at a time when all acute cocaine effects have subsided. This observed change therefore represents an enduring adaptation in the LHb, as it persists well beyond acute pharmacological effects of cocaine. However, the increase in LHb neuron excitability was not observed at a much later withdrawal time point of 45 days, indicating that it is not a permanent change. Thus, cocaine-induced adaptations in LHb neurons and any posited impact on negative affect appear to be reversible following a short-access cocaine regimen (2 hours per day for 5 days). Hence, there seems to be a window of time following cocaine exposure when LHb transmission is amplified. This window of amplified LHb signaling may be sufficient to trigger additional circuitry changes downstream, such as those found at LHb-to-RMTg synapses after cocaine exposure (Maroteaux and Mameli, 2012), particularly when considering the highly regulated and sensitive nature of LHb neurons (Wilcox et al., 1988; Weiss and Veh, 2011). The cocaine regimen used in this study was relatively mild, and a longer or stronger cocaine treatment regimen may result in larger or longer-lasting cellular effects relative to the results observed here.

Additional analysis was done to further examine the cause of the increased excitability in LHb neurons after cocaine self-administration. A number of membrane properties were measured, including the fast and medium components of AHP, the threshold of action potentials, and the membrane resistance of recorded cells from saline or cocaine treated animals. No differences were found between any of the examined cell characteristics, save for the membrane resistance in the cocaine ST and MT withdrawal group (Table 1; Figures 4d and 6d). Collectively, these results suggest that cocaine self-administration does not affect sodium channels or big/small conductance calcium-activated potassium channels of LHb cells and that these channels are not responsible for the increase in excitability. However, membrane resistance does appear to be correlated with an increase in excitability and can be affected by several factors—resting potassium channels being the principle mediators. Thus, if cocaine exposure were to increase dopamine signaling to LHb neurons by blocking reuptake, increased dopamine activity at LHb neurons (via D2 and D4 receptors) could hypothetically result in intracellular signaling cascades leading to a reduction in the number of passive potassium channels at the membrane that could persist for days or weeks before returning to baseline levels. There are other potential factors that could lead to increases in membrane excitability, but further investigation into the identities of the specific channels or receptors that might be involved was beyond the scope of the present study. We therefore cannot make definitive conclusions about the underlying mechanism by which cocaine self-administration may lead to increases in membrane excitability and resistance.

**Table 1.** Characteristics of LHb Regular Spiking (RS) Neurons 1 to 2, 5 to 7, and 45 Days after 5-Day Cocaine or Saline Self-Administration

RS LHb Cells		Current Steps (pA): Voltage difference (mV)					Current Steps (pA): Average Spike Number									Mem. Resist. (MΩ)	Threshold (mv)	mV from Threshold	
		-50	-40	-30	-20	-10	+10	+20	+30	+40	+50	+60	+70	+80	+90			fAHP (mV)	mAHP (mV)
Saline 1-2d	n=19	-27.47	-22.03	-18.00	-13.22	-7.32	3.00	4.40	6.72	8.14	9.50	10.26	11.47	12.74	13.58	618.60	-35.17	-23.50	-25.18
SEM		3.56	2.36	2.00	1.72	0.93	0.81	0.97	0.96	1.04	1.02	1.06	1.06	1.26	1.23	73.49	1.19	1.79	1.93
Cocaine 1-2d	n=19	-41.55	-33.96	-26.47	-19.07	-10.37	3.29	5.33	7.15	9.24	10.78	12.60	13.88	15.56	16.32	910.53	-34.04	-23.42	-27.13
SEM		5.25	3.73	3.10	2.55	1.58	0.50	0.50	0.58	0.69	0.76	0.92	0.94	1.07	1.08	115.91	1.07	1.70	1.83
Saline 5-7d	n=18	-34.89	-27.88	-20.32	-14.50	-7.79	2.75	4.89	6.04	7.46	8.62	9.65	10.95	12.22	13.27	632.37	-33.94	-24.43	-25.02
SEM		3.37	2.77	2.27	1.70	1.03	0.52	0.56	0.64	0.72	0.69	0.78	0.80	0.89	0.99	51.03	0.75	0.86	0.97
Cocaine 5-7d	n=19	-35.44	-30.45	-24.07	-17.34	-10.24	2.89	5.47	7.89	9.69	11.32	12.26	14.02	15.64	17.01	863.36 *	-36.00	-24.38	-24.75
SEM		2.88	2.59	1.87	1.36	1.02	0.36	0.45	0.54	0.51	0.59	0.68	0.81	0.87	1.11	72.13	0.99	1.21	0.93
Saline 45d	n=10	-35.43	-29.38	-20.89	-14.96	-8.74	2.50	4.33	6.05	7.55	8.60	10.15	11.30	12.57	13.47	732.42	-37.52	-23.84	-25.24
SEM		1.25	0.94	1.44	0.93	0.44	0.46	0.55	0.54	0.59	0.56	0.68	0.93	0.73	0.73	34.93	1.29	1.56	1.45
Cocaine 45d	n=15	-45.55	-36.44	-29.16	-20.16	-11.45	2.72	4.57	5.97	7.29	8.99	10.43	12.12	13.49	14.92	814.13	-36.46	-24.61	-25.57
SEM		2.87	2.95	2.43	2.13	1.40	0.29	0.48	0.62	0.67	0.83	0.96	1.07	1.24	1.31	52.57	1.20	1.29	1.59

Threshold denotes the voltage point when a cell’s voltage begins the final curve upwards to form the action potential peak at >25 mV/ms; bolded entry, *P* < .05 vs saline controls; *, *P* < .01 vs saline controls.

It is worth noting that all LHb neurons demonstrate clear rebound spiking following even brief and relatively weak hyperpolarizing current injections, as has been previously reported (Wilcox et al., 1988; Gutnick and Yarom, 1989; Chang and Kim, 2004; Weiss and Veh, 2011). This rebound spiking appeared to be dependent on the magnitude and length of the hyperpolarization stimulus and could last up to 30 seconds following a single hyperpolarizing period in some cases. This firing mechanism is especially interesting at the level of the circuit, because it potentially allows both incoming excitatory and inhibitory signals to elicit subsequent excitatory output from LHb neurons. This scenario has interesting implications regarding how plasticity develops within LHb pathways and how it may affect functional output and behavior. This question has apparently been underexplored to this point but seems to merit further investigation to determine the role of LHb rebound spiking in vivo. Interestingly, the rebound spiking patterns did not necessarily match the spiking patterns during depolarization within the same cell. Some cells that showed RS spike patterns during depolarizing current injections then showed BF rebound spiking patterns, despite resting at a constant membrane potential.

LHb neurons are known to fire spontaneously in slices. However, observed spontaneous tonic firing in LHb neurons was highly variable. Approximately 30% of patched cells would demonstrate no spontaneous tonic firing, approximately 40% of cells would initially show spontaneous tonic activity but would progressively lose that activity within 20 minutes, and approximately 30% of cells showed consistent spontaneous tonic firing activity lasting for 30+ minutes. When present, spontaneous tonic activity generally occurred at 0.1 to 10 Hz for standing spikes or at 0.1 to 2 Hz for tonic bursts. However, even in cells that showed consistent spontaneous tonic firing, the firing rate for an individual cell often fluctuated between higher and lower frequency over time. For these reasons, attempts to measure a reliable and consistent rheo base or an average tonic firing rate were deemed to be too volatile and inconsistent within the population of sampled LHb cells to make any reliable interpretations or conclusions. Other studies have noted similar levels of heterogeneity or variability regarding spontaneous firing in LHb cells (Chang and Kim, 2004; Weiss and Veh, 2011). No obvious differences in spontaneous tonic firing patterns or rates between LHb cells of cocaine- and saline-trained animals were apparent.

The VTA-to-LHb pathway is especially interesting, because the projecting neurons from the VTA have been shown to possess the TH marker for dopamine production while also possessing markers for GABA and glutamate release (Stuber et al., 2010; Stamatakis et al., 2013). There is evidence that these TH-positive VTA-to-LHb fibers do not actually release dopamine upon stimulation, but rather release GABA or glutamate (Stamatakis et al., 2013). On the other hand, cocaine has been shown to directly affect dopamine signaling within the LHb via D2 and D4 receptors (Good et al., 2013). The full picture of VTA-to-LHb signaling has yet to be revealed, but cocaine-induced changes to intrinsic membrane properties of LHb neurons would likely affect signaling sent along this peculiar pathway. These intrinsic membrane adaptations would broadly affect signal transduction at LHb neurons across all input pathways.

The results of the current study may also have implications beyond cocaine and addictive drug-use, because LHb activity also appears to modulate other reward behaviors such as sucrose intake (Friedman et al., 2011), presumably via similar pathways involving the RMTg and VTA. As the present study did not test rewarding stimuli other than cocaine, it is possible that LHb neurons have increased membrane excitability following other rewarding stimuli. In future experiments, it would be interesting to see how LHb excitability is affected by other drugs or sucrose compared to cocaine and saline.

Taken together, the present study characterizes LHb neurons in the parvocellular and central areas of the medial LHb at various time points after saline or cocaine self-administration and determines that LHb neurons have higher membrane excitability 1 to 7 days after cocaine self-administration. This effect returned to baseline by 45 days of withdrawal from cocaine. This change in cell excitability was correlated with changes in membrane resistance. As the LHb largely sends glutamatergic output to regions including the RMTg, which then sends GABAergic projections to the VTA, this result has potentially important implications in the sensitization of negative affect and opponent processes following addictive drug exposure (Solomon and Corbit, 1974; Koob and Le Moal, 2008), especially if this window of LHb signal amplification is sufficient to trigger additional long-lasting circuitry adaptations. Preventing or reversing these LHb neuron adaptations after cocaine exposure may assist in reducing chronic negative affect and reduce relapse.

## Statement of Interest

None.
